# Supplementary material for: Oxidative Stress Amelioration of Novel Peptides Extracted from Enzymatic Hydrolysates of Chinese Pecan Cake
Source: Int J Mol Sci. 2022 Oct 11;23(20):12086. doi: 10.3390/ijms232012086 (PMC9603611; doi:10.3390/ijms232012086)
Supplement: Supplementary file 1 [file ijms-23-12086-s001.zip › Supplementary.pdf]

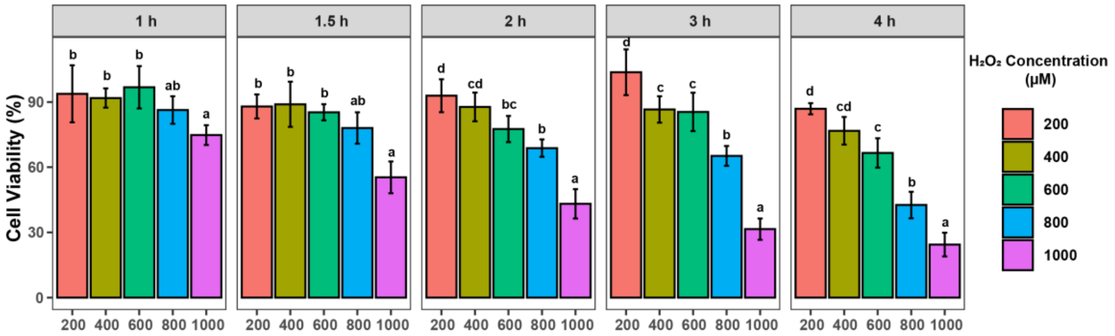

Figure S1. The influence of H_2O_2 at different combination of H_2O_2 concentration and treatment time. Different lowercases above the error bar denoted significant differences ($P < 0.05$).

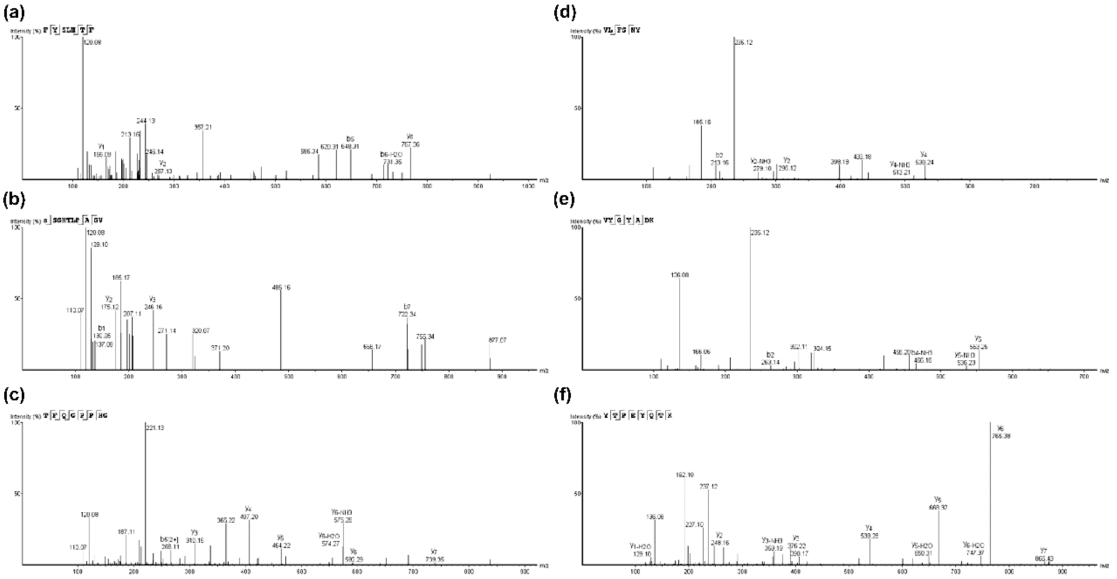

Figure S2. The MS/MS spectra (a-f) of the identified and synthesized peptides. (a) FYSLHTF; (b) SSGHTLPAGV; (c) TFQGPPHG; (d) VLFSNY; (e) VYGYADK; (f) YTPEYQTK.

Table S1. Column elution program

Time (min)	A (%)	B (%)
0	92	8
2	92	8
10	90	10
12	81	19
19	74	26
21	65	35
31	54	46
33	0	100
36	0	100
38	92	8
45	92	8

Table S2. Amino acids contents of ultrafiltration components (%)

Amino acid composition	Peptide fractions		
	PCPH–I (%)	PCPH–II (%)	PCPH–III (%)
Asp	10.39±0.28	11.40±0.25	11.42±0.52
Glu	26.21±0.54	22.15±0.90	14.94±0.01
Ser	5.20±0.07	5.80±0.08	6.56±0.03
Gly	4.39±0.17	4.50±0.09	4.82±0.05
His	2.03±0.06	1.80±0.09	1.96±0.05
Arg	5.34±0.16	5.01±0.15	4.89±0.01
Thr	3.68±0.12	3.94±0.09	3.92±0.01
Ala	6.84±0.36	6.59±1.66	6.10±0.17
Pro	4.65±0.03	6.58±1.77	8.03±0.32
Tyr	3.06±0.03	3.08±0.16	3.60±0.11
Val	5.05±0.28	5.41±0.38	6.28±0.12
Met	1.61±0.16	1.79±0.10	1.83±0.17
Cys	1.11±0.32	0.88±0.24	0.95±0.11
Ile	3.94±0.05	4.37±0.09	4.98±0.00
Leu	6.06±0.11	6.47±0.33	8.32±0.04
Phe	5.50±0.07	4.97±0.09	5.68±0.04
Lys	4.94±0.12	5.26±0.20	5.70±0.36
HAA	40.04±1.04	42.73±4.68	48.69±1.16
EAA	29.17±0.74	30.42±1.18	34.89±0.57
AAA	8.56±0.10	8.05±0.25	9.28±0.15

Asp: Aspartic acid; Glu: Glutamic acid; Ser: Serine; Gly: Glycine; His: Histidine; Arg: Arginine; Thr: Threonine; Ala: Alanine; Pro: Proline; Tyr: Tyrosine; Val: Valine; Met: Methionine; Cys: Cysteine; Ile: Isoleucine; Leu: Leucine; Phe: Phenylalanine; Lys: Lysine.

Hydrophobic amino acids (HAA)=Ala, Pro, Tyr, Val, Met, Ile, Leu, Phe;

Essential amino acid (EAA)=Thr, Tyr, Val, Met, Ile, Leu, Phe, Lys

Aromatic amino acids (AAA)=Phe, Tyr.

PCPH−I (>10kDa), PCPH−II(3-10kDa) and PCPH−III (<3kDa).
